# Influence of Depression and Anxiety on Non-Surgical Periodontal Treatment Outcomes: A 6-Month Prospective Study

**DOI:** 10.3390/ijerph18179394

**Published:** 2021-09-06

**Authors:** Catherine Petit, Victor Anadon-Rosinach, Nicolas Tuzin, Jean-Luc Davideau, Olivier Huck

**Affiliations:** 1 Faculté de Chirurgie Dentaire, Periodontology, Université de Strasbourg, 67000 Strasbourg, France; catherin.petit@gmail.com (C.P.); victor.anadonrosinach@gmail.com (V.A.-R.); jldcabfra@wanadoo.fr (J.-L.D.); 2 Pôle de Médecine et Chirurgie Bucco-Dentaires, Hopitaux Universitaires de Strasbourg, 67000 Strasbourg, France; 3 INSERM (French National Institute of Health and Medical Research), UMR 1260, Regenerative Nanomedicine, Fédération de Médecine Translationnelle de Strasbourg (FMTS), 67000 Strasbourg, France; 4 Groupe Méthode en Recherche Clinique, Service de Santé Publique, Hôpitaux Universitaires de Strasbourg, 67000 Strasbourg, France; nicolas.tuzin@chru-strasbourg.fr

**Keywords:** periodontitis, depression, anxiety, periodontal treatment

## Abstract

Periodontal treatment could be worsened by risk factors. Depression and anxiety have been suggested as potentially influencing periodontal treatment outcomes. The aim of this study was to determine their association with non-surgical periodontal treatment outcomes in patients with generalized severe periodontitis (stage III/IV generalized periodontitis) at 6 months. A total of 68 patients diagnosed with generalized severe periodontitis were treated with scaling and root planing (SRP) and were followed at 3 and 6 months. The data of the 54 patients that followed the entire protocol were considered for analysis. Depression and anxiety levels were determined at baseline by the Beck Depression Inventory (BDI) and State-Trait Inventory (STAI) questionnaires. The association between psychological scores and periodontal parameters was evaluated by multivariate analysis. At 3 and 6 months, SRP induced an improvement for all periodontal parameters (plaque index (PI), bleeding on probing (BOP), periodontal probing depth (PPD) and clinical attachment loss (CAL)). BDI and STAI scores were associated with the evolution of PI, BOP, mean PPD and number of sites with PPD > 3 mm and with CAL > 3 mm. Depression and anxiety should be considered as risk factors for SRP and the identification of at-risk patients should be performed using well-established tools.

## 1. Introduction

Periodontitis is a chronic inflammatory disease induced by oral dysbiosis and characterized by clinical symptoms, including gingival swelling, gingival bleeding, clinical attachment loss (CAL) and tooth mobility [1]. This highly prevalent disease impacts the oral-health-related quality of life and its long-term management is at a high cost [2,3]. The development of periodontitis involves the disruption of the balance between the host response and polymicrobial communities promoted by keystone pathogens, such as *Porphyromonas gingivalis* [4]. Periodontal pathogens will invade the gingival tissues and elicit an exacerbated inflammatory response mediated by cytokines and chemokines [5,6]. Such an inflammatory process will modulate the bone homeostasis through the promotion of osteoclastogenesis, contributing to the destruction of the alveolar bone [7]. Interestingly, several factors have been identified as risk factors or risk indicators, such as smoking, systemic diseases and, more recently, psychological factors [8,9,10,11]. Indeed, several inflammatory mediators (IL-1b, IL-6 and IL-8) implicated in the development of periodontal diseases have correlated with the level of stress, emphasizing a link between periodontal status and psychological status [8,10,12,13].

Currently, the treatment of periodontitis is mainly non-surgical (scaling and root planing (SRP)) and aims to remove oral biofilms and to restore homeostasis between bacterial commensals and the host response [14]. Several factors can also negatively influence SRP outcomes. While smoking and chronic diseases, such as diabetes, are the most described factors [15,16,17], the psychological status has also been suggested as worsening SRP outcomes [10,18,19,20,21,22]. Indeed, it was observed that patients with increased stress, anxiety and depression scores, as well as those exhibiting negative coping strategies, demonstrate worsened SRP outcomes [18,19,20]. Depression represents a major health concern, as it was shown that depressed patients are more affected by chronic inflammatory diseases, such as diabetes [23,24]. In the context of periodontal diseases, it was observed that the incidence of depression was higher in periodontitis patients, highlighting a potential relationship between both conditions [20,25,26]. The same type of association was depicted regarding the prevalence of anxiety in patients affected by chronic inflammatory diseases, including periodontitis [27,28]. However, some contradictory results could be found within the scientific literature [29]. Such discrepancies could be explained by the specific type of population investigated and the use of different methods of psychological evaluation. Indeed, several questionnaires are commonly used to determine depression or anxiety, such as the Depression Anxiety Stress Scale (DASS-42), State-Trait Anxiety Inventory (STAI) or Beck Depression Inventory (BDI). DASS-42 is a self-reported questionnaire that evaluates the negative emotional states of depression, anxiety and stress. Each of the three DASS-scales contain 14 items. Indeed, the depression scale assesses dysphoria, hopelessness, the devaluation of life, self-deprecation, lack of interest/involvement, anhedonia and inertia. The essential function of the DASS is to assess the severity of the core symptoms of depression, anxiety and stress [30]. The STAI is a commonly used measure of trait and state anxiety used in clinical settings to diagnose anxiety and to distinguish it from depressive syndromes [31]. It consists of 20 items assessing trait anxiety and 20 items assessing state anxiety. BDI is a 21-item questionnaire that measures characteristic attitudes and symptoms of depression [32].

As the tools used to characterize the association between psychological status and periodontal treatment outcomes are diverse, the aim of this study was to determine the influence of depression and anxiety assessed by self-administered questionnaires i.e., STAI and BDI, on SRP outcomes at 6 months in a French population suffering from severe chronic periodontitis (stage III–IV generalized periodontitis).

## 2. Materials and Methods

### 2.1. Study’s Protocol

This is an additional analysis of a study conducted in accordance with the Declaration of Helsinki and approved by the local ethical committee (Comité de protection des personnes 3/30; clinical trials: NCT02568163) [11]. All participants were enrolled at the Department of periodontology, University hospital, Strasbourg, France and informed about the protocol and aim of the study, and gave written consent prior to entering the study.

### 2.2. Inclusion Criteria

As described previously, adult patients (>18 years old) diagnosed with generalized severe chronic periodontitis [33] (stage III-IV generalized periodontitis) characterized by alveolar bone loss assessed on radiographs, CAL > 4 mm, more than 15 teeth with at least 5% of the sites with PPD > 5 mm and radiographic bone loss were included [11]. Patients with systemic diseases (diabetes, auto-immune diseases, chronic inflammatory diseases, etc.) and/or treated with medications that could affect periodontium, such as antibiotics and anti-inflammatory or psychotropic drugs, in the last 6 months, were excluded. Patients that were treated with specialized periodontal treatment in the last 6 months, as well as pregnant women and patients wearing orthodontic appliances, were also excluded. Patients that did not attend recall, having used antibiotics or anti-inflammatory drugs, were excluded from the study.

### 2.3. Psychological Measurements

**Figure 1:**
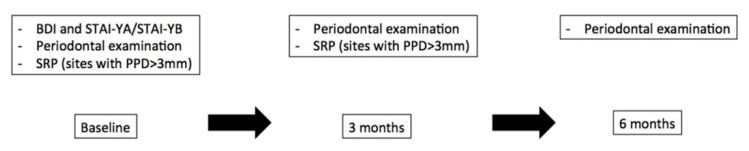
Flow chart of the study.

Psychological status was evaluated using self-administered questionnaires. The French versions of BDI and STAI-YA/STAI-YB questionnaires were filled by patients at baseline. BDI questionnaire is a 21-item questionnaire that requires 10 min to be filled. This questionnaire allows for the quantitative evaluation of the intensity of the depressive feelings. STAI has 20 items for assessing trait anxiety and 20 for state anxiety, each item being scored on a 4-point scale, and requires 10 min to be filled. Questionnaire’s scores were blinded to both patients and investigators in charge of the periodontal examination and SRP (Figure 1).

### 2.4. Non-Surgical Periodontal Treatment and Follow-Up

Periodontal indexes (PPD, CAL, PI and BOP) were collected prior to the treatment using a PCPUNC 15 periodontal probe (Hu-Friedy, Chicago, IL, USA) at baseline, 3 and 6 months. Patients were treated as follows: oral hygiene instructions, SRP at sites with PPD > 3 mm using ultrasonic devices (Suprasson Newtron, Satelec, France) and manual curets (Deppeler, Switzerland) under local anesthesia, in 2 sessions within 7 days. Following each session, patients were instructed to rinse with chlorhexidine (0.12%) mouthwash (Eludril, Pierre Fabre, Cahors, France) for 15 days. At 3 months, residual sites with PPD > 3 mm were re-instrumented. All periodontal examinations, as well as SRP, have been performed by the same operator blinded to psychological measurements results.

### 2.5. Statistical Analysis

The assessment of examiner reliability showed that more than 90% of repeated measurements were within +/−1 mm of the original, indicating good intra-examiner reliability and agreement (kappa score > 0.9). For an alpha risk of 5% and a power of 80%, the number of subjects required to obtain a correlation coefficient of 0.4 is 47. By adding 15% in case of possible drop out, the minimal number of subjects required is equal to 54 patients [20]. All interactions were tested by the Wald procedure in generalized linear models. Influencing parameters with a *p*-value < 0.2 were considered in a multivariable model. All statistical tests were two-tailed. A *p*-value < 0.05 was considered statistically significant. All analyses were performed using R software under its version 3.0 (R Core Team (2014). R: A language and environment for statistical computing. Vienna, Austria).

## 3. Results

### 3.1. Study’s Sample and Demographics

Among the 68 patients fulfilling the inclusion criteria that were included in this study, 54 completed the 6 months of follow-up. The main reasons for exclusion were the non-respect of the study’s protocol, especially any cancelled or delayed follow-up sessions. The mean age of the sample population was 51.2 years old (SD = 10.1) and 56% of the sample population was female. Nevertheless, 39% of the patients were smokers, with 17 patients consuming more than 10 cigarettes/day and 26% being an ex-smoker. Seventeen percent were consuming alcohol more than 1 time/day. Seventy percent of the patients were married and 72% were employed. Regarding the psychological status of the patients, the mean score of BDI was 9.6 (6.7), 37.9 (10.8) for STAI-YA and 40.8 (10.4) for STAI-YB.

### 3.2. Non-Surgical Periodontal Treatment Outcomes

**Figure 2:**
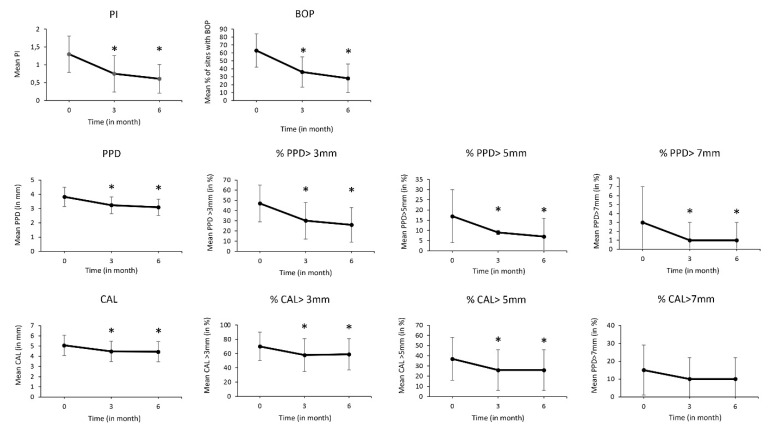
Evolution of periodontal parameters following SRP. PI, mean % of sites with BOP, mean PPD (mm), % of sites with PPD > 3 mm, >5 mm, 7 mm, mean CAL (mm), % sites with CAL > 3 mm, >5 mm, 7 mm were measured at baseline, 3 and 6 months. *: *p* < 0.05 vs. baseline.

After 6 months, SRP was effective in significantly reducing PI (−0.7 (0.11)), BOP (−35% (11)), mean PPD (−0.7 mm (0.1)) and the number of shallow (PPD > 3 mm) (−27.4 (17.7)) and deep sites (PPD > 5 mm) (−12.2 (12)) (*p* < 0.05) (Figure 2). Interestingly, age, gender, alcohol consumption and smoking habits negatively influenced SRP outcomes (*p* < 0.05) and, therefore, such parameters were considered in our analysis in order to determine the association between the BDI and STAI scores and SRP outcomes (Table 1). Interestingly, it was estimated that the BDI score was associated with the evolution of PI (*p* < 0.001), PPD > 3 mm (*p* < 0.001) and ΔCAL > 3 mm (*p* = 0.03). Additionally, STAI-YA was associated with ΔBOP (*p* < 0.001) and ΔPPD (mean) (*p* < 0.05), whereas STAI-YB was associated with ΔBOP (*p* < 0.001) only.

## 4. Discussion

Psychological status has been demonstrated as a risk factor for worsened periodontal treatment outcomes. In this study, a specific focus was made on depression and anxiety, which were evaluated using BDI and STAI self-administered questionnaires, and associations between the score and SRP outcomes were observed. This confirms that several questionnaires can be used by dentists to detect at-risk patients prior to SRP.

Psychological factors have been suggested as being associated with the increased prevalence of periodontitis due to the inflammatory involvement of the diseases, but also due to the impact on the patients’ behaviors [34,35]. However, this hypothesis remains under discussion, as, in some studies, no differences were observed between the healthy population and patients diagnosed with psychological disorders, such as severe depression [36]. A recent meta-analysis pointed out that that subjects with periodontal disease had a higher depression scale score and anxiety score; however, the heterogeneity between studies highlighted the necessity of additional ones [37]. Indeed, the characterization of depression could be difficult, as several questionnaires are commonly used.

In this study, we aimed to evaluate the impact of depression and anxiety assessed with STAI and BDI questionnaires on periodontal treatment outcomes. The French versions of STAI and BDI were selected due to their strong validation, reliability, internal consistency and extensive use in order to evaluate the association between psychological status and chronic diseases [38,39,40,41]. In a previous analysis where depression level was measured using the depression score of the DASS-42 self-administered questionnaire, an association between depression and worsened SRP outcomes was observed, especially regarding the evolution of BOP and reduction in deep sites with PPD > 5 mm and >7 mm [20]. When the patients’ depression status was evaluated using BDI, a negative association with the SRP outcomes for PI, PPD > 3 mm and CAL > 3 mm was measured. It was previously observed that the depression component of DASS was correlated with the BDI scores [42]. Here, it should be mentioned that different periodontal parameters were influenced by each questionnaire’s score. This may be explained by the distribution of the patient’s scores on each questionnaire’s scale, and the limited number of patients with high scores of depression. Regarding anxiety, the STAI questionnaire was used to determine the state and trait of anxiety of the patients. It was observed in several clinical studies that anxiety was related to periodontitis prevalence and severity. It is worth noting that anxiety can lead to behavioral changes that might worsen treatment outcomes, or, in a worst case scenario, lead to treatment interruption [43,44,45,46]. In a murine model of ligature-induced periodontitis, the presence of anxiety-like behaviors was observed in the acute phase of periodontitis, highlighting a potential role for such a psychological trait or state in the pathological process [47]. Here, the association between STAI scores and periodontal parameters evolution was observed. However, it would have been of interest to precisely determine the influence of the anxiety level on the oral hygiene habits of the patients in order to assess the potential modification on the patient’s behavior and its impact on SRP outcomes. For instance, psychological diseases will be associated with the adoption of health-damaging behaviors, such as tobacco smoking or increased alcohol consumption, inadequate sleep quality, poor diet choices, insufficient hygiene practice or a poor compliance with appointments/postoperative treatment suggestions [48]. Such an observation was observed for an instance in a population of stressed police recruits, where patients with a high level of stress presented more plaque accumulation [49].

Several mechanisms have been suggested to link depression and anxiety to periodontitis. Indeed, neuroinflammation sustained by the chronic inflammation associated with periodontitis, increased systemic lipopolysaccharide and bacterial translocation from periodontal sites through a more permeable blood–brain barrier has been investigated [50,51]. The development of depression-like behavior with brain modifications was observed in mice with experimental periodontitis induced by oral gavage of Porphyromonas gingivalis, such as an increased number of activated astrocytes and decreased levels of mature brain-derived neurotrophic factor and astrocytic p75NTR in the hippocampus [52]. Nevertheless, during periodontitis, cytokines, such as Il-1β, IL-6 and TNF-α, are secreted in response to bacterial insult and could sustain the neuroinflammation process after passage through the blood–brain barrier [34,50]. Such increased levels of cytokines could be measured in the gingival crevicular fluid of depressed patients, as observed in a cohort of depressed women on long-term sick leave [53] and women with stress-related depression [54]. However, these changes vary according to the severity of both periodontitis and psychological disease [55]. A depression state is also associated with a decreased immune response, characterized by decreased leukocyte mobilization, decreased innate and adaptative responses, increased Th1 and Th2 response and decreased immunoprotection, altogether contributing to an increased susceptibility to infection and delayed wound healing [48].

The present study presents some limitations. Firstly, as only patients with severe forms of periodontitis were enrolled, the magnitude of the influence of risk factors, such as depression and anxiety, on SRP outcomes may have been blunted by local factors. In addition, only a reduced number of patients presented high levels of depression or anxiety, and large studies with more patients with different levels of depression or anxiety should be conducted in order to determine clear thresholds for an increased risk. We also decided to include smokers, even if smoking is a well-described risk factor for worsened periodontal treatment outcomes. This strategy was selected in order to avoid excluding patients that potentially exhibit a high level of anxiety or depression. This could be considered as a limitation, and future studies may focus exclusively on non-smoker or smoker populations.

## 5. Conclusions

In conclusion, it can be mentioned that BDI and STAI questionnaires could be proposed as interesting tools to evaluate the risk of worsened SRP outcomes associated with psychological status. These quick and reliable questionnaires could be used by dentists. This strategy may help to detect patients in need of specialized psychological consultation and to adapt periodontal treatment strategy. For patients exhibiting psychological diseases, short periodontal maintenance recalls will be of interest in order to maintain periodontal health through constant motivation of the patients and the reinforcement of oral hygiene procedures. During the active phase of the treatment, the use of adjunctive therapies may be of interest (antibiotics, antiseptics), especially in immune-compromised patients [48]. However, the impact of such modified treatment procedures should be evaluated in future studies.

## Figures and Tables

**Table 1:** Multivariate analysis of covariance between periodontal treatment outcomes and psychological scores. Associations were analyzed by linear and logistic mixed regression models considering psychological parameters at baseline and the evolution of periodontal parameters between baseline and 6 months. All potential influencing variables were considered (age, smoking, gender) in the mathematical model. Significant associations are in bold (*p* < 0.05). ΔPI, ΔBOP, ΔPPD and ΔCAL represent the difference for mean PI, BOP, PPD and CAL between baseline and 6 months, respectively; ΔPPD > 3 mm, ΔPPD > 5 mm and ΔPPD > 7 mm represent the difference for number of sites with PPD > 3 mm, >5 mm and 7 mm between baseline and 6 months. ΔCAL > 3 mm, ΔCAL > 5 mm and ΔCAL > 7 mm represent the difference for number of sites with PPD > 3 mm, >5 mm and 7 mm between baseline and 6 months.

Variable	Score	Estimate (SD)	OR (CI)	*p*-Value
ΔPI	BDI	−0.01 (0.003)		<0.001
	STAI-YA	−0.00 (0.002)		0.96
	STAI-YB	0.01 (0.002)		<0.001
ΔBOP	BDI	0.00 (0.003)		0.91
	STAI -YA	0.019 (0.002)		<0.0001
	STAI-YB	−0.017 (0.002)		<0.0001
ΔPPD (mean)	BDI	−0.010 (0.006)		0.070
	STAI-YA	0.010 (0.004)		0.014
	STAI-YB	−0.006 (0.004)		0.202
ΔPPD > 3 mm	BDI		0.97 (0.95–0.99)	0.004
	STAI-YA		1.01 (0.99–1.02)	0.113
	STAI-YB		0.999 (0.98–1.01)	0.874
ΔPPD > 5 mm	BDI		1.011 (0.98–1.04)	0.435
	STAI-YA		1.01 (0.99–1.03)	0.290
	STAI-YB		0.99 (0.97–1.01)	0.584
ΔPPD > 7 mm	BDI		1.03 (0.97–1.10)	0.321
	STAI-YA		1.01 (0.97–1.05)	0.523
	STAI-YB		0.98 (0.93–1.02)	0.282
ΔCAL (mean)	BDI	−0.014 (0.008)		0.075
	STAI-YA	0.003 (0.005)		0.637
	STAI-YB	0.007 (0.006)		0.272
ΔCAL > 3 mm	BDI		0.980 (0.96–1.00)	0.03
	STAI-YA		1.007 (0.99–1.02)	0.453
	STAI-YB		1.007 (0.99–1.02)	0.332
ΔCAL > 5 mm	BDI		0.99 (0.97–1.00)	0.344
	STAI-YA		1.002 (0.98–1.02)	0.787
	STAI-YB		1.008 (0.99–1.02)	0.268
ΔCAL > 7 mm	BDI		0.99 (0.96–1.01)	0.486
	STAI-YA		1.003 (0.98–1.02)	0.785
	STAI-YB		1.006 (0.98–1.02)	0.552

## Data Availability

Data will be available from authors upon reasonable request.
